# *Mycoplasma phocimorsus* (mῑ-kō-′plaz-mǝ fō-ki-′mȯr-sǝs), panaritium (pan-ə-′rish-ē-əm)

**DOI:** 10.3201/eid3102.241778

**Published:** 2025-02

**Authors:** Clyde Partin

**Affiliations:** Emory University School of Medicine, Atlanta, Georgia, USA

**Keywords:** Mycoplasma phocimorsus, panaritium, bacteria, Denmark

This issue of EID incudes a report of patient with a cat scratch–induced panaritium caused by infection with the bacterium *Mycoplasma phocimorsus* (see page 380). In 2023, researchers at Statens Serum Institut in Denmark reported a novel species of the bacterial class Mollicutes (from the Latin *mollis* for soft and *cutis* for skin). The new species was named *Mycoplasma phocimorsus* (*phoca* for seal, *morsus* for bite); 6 strains were found in samples from Denmark, Norway, and Sweden.

The associated infection, first described in 1907, was called seal, blubber, or spekk (Norwegian for blubber) finger because infected persons had been exposed to seals in marine environments. The superficial lesions around the fingernail are called whitlows, but deeper penetration involves the tendon sheath, a painful condition deemed panaritium tendineum. (Panaritium, used interchangeably with whitlow or paronychia, more correctly implies purulent inflammation and infection of digital tendons.)

The etymology for *Mycoplasma* (*mykes* for fungus, *plasma* for formed) is complicated. The word was introduced by A.B. Frank in 1889 to denote an intimate relationship between plant-invading fungi or other microorganisms and their host cells, whose cytoplasm is altered by the infection. Frank described mycoplasma as a “mixture of fungal and plant protoplasm…that it gave rise to bacteroid tissue.”

**Figure:**
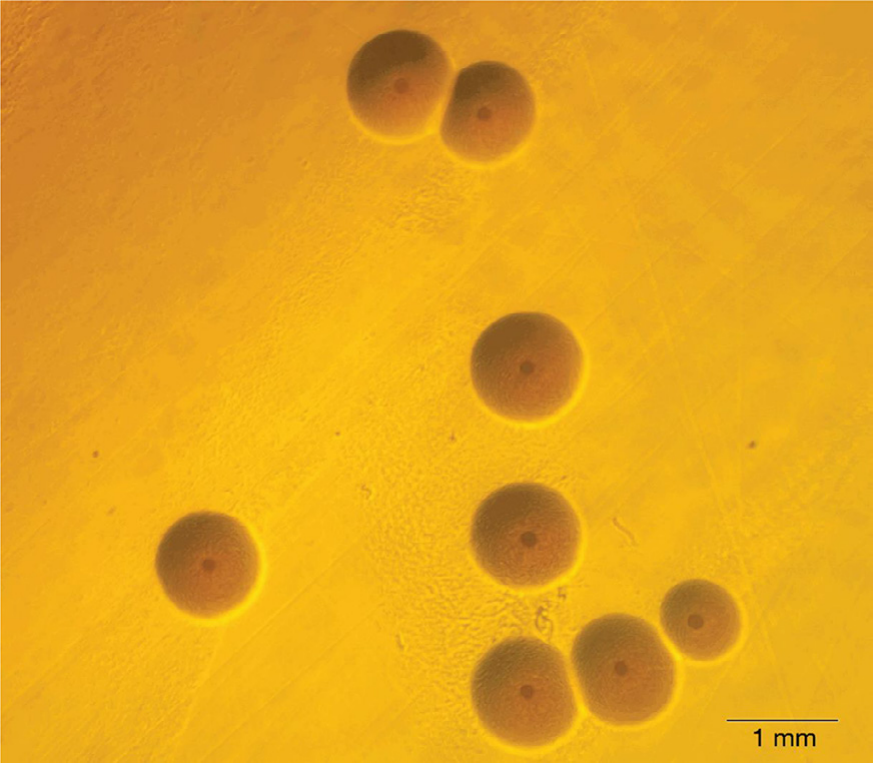
*Mycoplasma phocimorsus*. Copyright ©2023 International Journal of Systematic and Evolutionary Microbiology. Used with permission.
